# Amino Acid-Based Metabolic Profile Provides Functional Assessment and Prognostic Value for Heart Failure Outpatients

**DOI:** 10.1155/2019/8632726

**Published:** 2019-05-19

**Authors:** Chao-Hung Wang, Mei-Ling Cheng, Min-Hui Liu, Tieh-Cheng Fu

**Affiliations:** ^1^Heart Failure Research Center, Division of Cardiology, Department of Internal Medicine, Chang Gung Memorial Hospital, Keelung, Taiwan; ^2^Chang Gung University College of Medicine, Taoyuan, Taiwan; ^3^Metabolomics Core Laboratory, Healthy Aging Research Center, Chang Gung University, Taoyuan, Taiwan; ^4^Department and Graduate Institute of Biomedical Sciences, College of Medicine, Chang Gung University, Taoyuan, Taiwan; ^5^Clinical Metabolomics Core Laboratory, Linkou Chang Gung Memorial Hospital, Taoyuan, Taiwan; ^6^Department of Nursing, National Yang-Ming University, Taipei, Taiwan; ^7^Department of Physical Medicine and Rehabilitation, Chang Gung Memorial Hospital, Keelung, Taiwan; ^8^Graduate Institute of Clinical Medical Sciences, College of Medicine, Chang Gung University, Taoyuan, Taiwan

## Abstract

Functional capacity is a crucial parameter correlated with outcomes. The currently used New York Heart Association functional classification (NYHA Fc) system has substantial limitations, leading to inaccurate classification. This study investigated whether amino acid-based assessment on metabolic status provides an objective way to assess functional capacity and prognosis in heart failure (HF) outpatients. Plasma concentrations of histidine, ornithine, and phenylalanine (HOP) were measured on 890 HF outpatients to assess metabolic status by calculating the HOP score. Cardiopulmonary exercise testing (CPET) was performed in 387 patients to measure metabolic equivalents (MET) in order to define the functional class based on MET (MET Fc). Patients were followed for composite events (death/HF-related rehospitalization) up to one year. We found only 47% concordance between the MET Fc and NYHA Fc. HOP scores worked better than NYHA Fc for discriminating patients with MET Fc II and III from those with MET Fc I, with the optimal cutoff value set at 8.8. HOP scores ≥ 8.8 were associated with risk factors for composite events in different kinds of HF populations and were a powerful predictor of composite events in univariate analysis. In multivariable analysis, HOP scores ≥ 8.8 remained a powerful event predictor, independent of other risk factors. Kaplan-Meier curves revealed that HOP scores of ≥8.8 stratified patients at higher risk of composite events in a variety of HF populations. In conclusion, amino acid-based assessment of metabolic status correlates with functional capacity in HF outpatients and provides prognostic value for a variety of HF populations.

## 1. Introduction

Heart failure (HF) is becoming a tremendous burden on healthcare systems worldwide. Functional capacity is a crucial parameter correlated with outcomes [1–4]. Currently, the gold standard for assessing the functional state is the cardiopulmonary gas exchange exercise test (CPET) [5, 6]. Because it requires instruments and is inconvenient to administer because it is time-consuming, the New York Heart Association functional classification (NYHA Fc) is widely used instead [7]. However, previous studies found that interobserver reproducibility of NYHA Fc when assessing class II and class III was only 56%, a result little better than chance [4]. A more precise assessment tool is needed.

Functional assessment estimates the severity of imbalance between cardiac supply and whole body demand, which can represent the entire body's metabolic status. Previously, we and others demonstrated that patients' plasma-based metabolic profile provided valuable information about HF-related metabolic disturbance [8–10], diagnosis [11–13], and prognosis [11, 14, 15]. We subsequently simplified the metabolomics assessment into an amino acid-based profile that includes histidine, ornithine, and phenylalanine (HOP score) [16, 17]. We found that the HOP score was well-correlated with functional capacity, as estimated by a six-minute walking distance.

Although NYHA functional classes III and IV suggest poor outcomes, the largest group of outpatients is usually in the NYHA class II category, which is often overlooked by clinicians. However, results of recent clinical trials strongly recommend active intervention for all patients from classes II to IV [1, 2]. In this study, we would like to use CPET to investigate whether HOP scores could be an objective substitute for identifying HF outpatients in the functional class ≥ II. We also would like to see whether the HOP-defined worse functional classification represents higher risk of HF-related rehospitalization/death in 12 months among HF patients with reduced ejection fraction (HFrEF), mid-range EF (HFmrEF), and preserved EF (HFpEF) [18].

## 2. Methods

### 2.1. Patients and Study Design

From January 2014 to May 2017, patients were enrolled consecutively at an outpatient HF clinic based on these inclusion criteria: (1) had been hospitalized due to acute or decompensated chronic HF, (2) at least one month after discharge, (3) NYHA functional class ≤ III, and (4) ages 20 to 85 years old.

Exclusion criteria included (1) the presence of disorders other than HF that might compromise survival within 6 months; (2) patients being bed-ridden for >3 months; (3) the presence of systemic diseases such as hypothyroidism, decompensated liver cirrhosis, and systemic lupus erythematosus; (4) patients with severe coronary artery disease without complete revascularization therapy; and (5) patients with a serum creatinine of >3 mg/dl. Informed consent was obtained from all patients. The study was designed and carried out in accordance with the principles of the *Declaration of Helsinki* and with approval from the Ethics Review Board of Chang Gung Memorial Hospital.

### 2.2. Blood Sampling and Examination

Blood samples for the metabolic panel were collected in EDTA-containing tubes on the day of the HF outpatient clinic in the early morning, after fasting for eight hours. For patients undergoing CPET, blood samples were collected within seven days before or after the CPET. Plasma was analyzed by UPLC workflow. BNP was measured in triplicate with the Triage BNP Test (Biosite, San Diego, CA), which was a fluorescence immunoassay for quantitative determination of plasma BNP. Precision, analytical sensitivity, and stability characteristics of the assay were previously described [19]. The measurement of other parameters, including estimated glomerular filtration rate (eGFR), hemoglobin, and albumin, was conducted in the central core laboratory.

### 2.3. Ultraperformance Liquid Chromatography (UPLC)

HOP scores were calculated as described in our previous study based on the values of histidine, ornithine, and phenylalanine, measured by UPLC [16]. EDTA plasma samples were collected and stored at −80°C until assayed. The plasma samples (100 *μ*l) were precipitated by adding an equal volume (100 *μ*l) of 10% sulfosalicylic acid containing an internal standard (norvaline, 200 *μ*M). After protein precipitation, the samples were vortexed and centrifuged at 12,000g for 10 min at room temp. After the samples were centrifuged, 20 *μ*l of the supernatant was mixed with 60 *μ*l working buffer (borate buffer, pH 8.8). The derivatization was initiated by the addition of 20 *μ*l of 10 mM AQC in acetonitrile. After 10 min incubation, the mixture was added with an equal volume of Eluent A (20 mM ammonium formate/0.6% formic acid/1% acetonitrile) and analyzed using the ACQUITY UPLC System [20, 21]. AQC derivatization reagent was obtained from Waters Corporation (Milford, MA, USA). An aqueous amino acid standard mixture was prepared at different concentrations (0, 25, 50, 100, 250, and 500 *μ*M) for each amino acid and was done by the same procedure. The Waters ACQUITY UPLC System consisted of a Binary Solvent Manager (BSM), a sample manager fitted with a 10 *μ*l loop, and a Tunable UV (TUV) detector. The system was controlled and data collected using Empower™ 2 Software. Separations were performed on a 2.1 × 100 mm ACQUITY UPLC BEH C18 column at a flow rate of 0.70 ml/min. The average intra-assay coefficient of variation was 4.3% for histidine, 4.6% for ornithine, and 4.6% for phenylalanine. A total coefficient of variation was 3.1% for histidine, 3.6% for ornithine, and 3.7% for phenylalanine. The detection limit was 0.5 *μ*M for histidine, 2.0 *μ*M for ornithine, and 3.3 *μ*M for phenylalanine. The linear range was 25–500 *μ*M for these three amino acids.

### 2.4. Cardiopulmonary Exercise Test

From January 2014 to May 2017, 387 patients performed graded exercise on a bicycle ergometer (150P; Ergoselect, Lindenstrasse, Germany) to evaluate their aerobic fitness and hemodynamic function. We correlated the peak metabolic equivalent (MET) (${\dot{\text{V}}}_{{\text{O}}_{2\text{peak}}}$) on the test with the HOP scores. The exercise test was carried out in an air-conditioned laboratory with an atmospheric temperature of 22–25°C, a barometric pressure of 755 to 770 Torr, and a relative humidity of 55–65%. The exercise protocol comprised 2 min of unloaded pedaling followed by a continuous increase of work rate of 10 W every minute until exhaustion (progressive exercise to peak oxygen consumption, ${\dot{\text{V}}}_{{\text{O}}_{2\text{peak}}}$). Oxygen consumption (${\dot{\text{V}}}_{{\text{O}}_{2}}$) was measured breath by breath using a computer-based system (MasterScreen CPX; Cardinal Health, Hoechberg, Germany). Heart rate was determined from the R-R interval on a 12-lead electrocardiogram, blood pressure was measured by an automatic blood pressure system (Tango; SunTech Medical, Eynsham, UK), and arterial O_2_ saturation was monitored by finger pulse-oximetry (model 9500; Nonin Onyx, Plymouth, MN). The ${\dot{\text{V}}}_{{\text{O}}_{2\text{peak}}}$ was defined by the following criteria: (1) the level of ${\dot{\text{V}}}_{{\text{O}}_{2}}$ increases less than 2 ml/kg per minute over at least 2 min, (2) heart rate exceeds 85% of its predicted maximal value, (3) the respiratory exchange ratio exceeds 1.15, or (4) some other symptom/sign limitations, according to the guidelines of the American College of Sports Medicine for exercise testing. Since the peak METs calculated for the bicycle exercise were 80% of the peak METs calculated for the treadmill exercise [22, 23], MET Fc I, II, and III were defined as peak MET ≥ 5.6, ≥4, and <4, respectively, as determined by bicycle exercise.

### 2.5. Follow-Up Program

Follow-up data were prospectively obtained every month from hospital records, personal communication with the patients' physicians, telephone interviews with patients, and patients' regular visits to staff physician outpatient clinics (followed for up to one year until they died, the study ended, or they were lost to follow-up). “Rehospitalization” was defined as HF-related rehospitalizations. A committee of three cardiologists adjudicated all hospitalizations without knowledge of patients' clinical variables to determine whether the events were related to worsening HF. “All-cause death” was chosen as an endpoint because of the interrelationship of HF with other comorbidities in the patient cohort. The composite event of HF-related rehospitalization and all-cause death (time to the first event) was analyzed for prognostic purposes.

### 2.6. Statistical Analyses

Results are expressed as the mean ± SD for variables with normal distribution and the median (interquartile range (IQR)) for variables with skewed distribution. Categorical variables are presented as numbers (percentages). Data were compared by two-sample *t*-tests, ANOVA (subgroup analysis was conducted by Bonferroni correction), and chi-square (multiple comparison with Bonferroni-adjusted *p* values) when appropriate. We estimated receiver operating characteristic (ROC) curves and used Youden's index to identify the cutoff values of variables. We used C-statistic to compare the diagnostic values between curves. Follow-up data were collected as scheduled or at the last available visit. We used Cox proportional hazard models to adjust the independent value of the HOP score in predicting the first defined events (death or HF-related rehospitalization). Variables with *p* value < 0.05 in the univariate analysis were selected for the multivariable analysis. Hazard ratios (HRs) and 95% confidence intervals (CIs) were calculated. To compare the time-dependent outcomes, we performed Kaplan-Meier analyses with a log-rank test. All statistical analyses were two-sided and performed using SPSS software (version 22.0, SPSS, Chicago, IL, USA) and R software (version 3.5.1). A *p* value of < 0.05 was considered significant.

## 3. Results

### 3.1. Baseline Characteristics and Laboratory Data among Patients with Differing MET Fc

CPET and HOP measurements were conducted on 387 patients. Based on the peak MET, patients were divided into three groups, including MET Fc I, II, and III. There was a significant trend associating higher MET Fc with older patients, less often male, who had higher LVEF, heart rate, and BNP levels. Higher MET Fc was also linked to higher incidence of diabetes mellitus, ischemic etiology, and use of diuretics, along with a wider QRS complex. Higher MET Fc was associated with lower body mass index, hemoglobin, albumin, and eGFR. We found only 47% concordance between MET Fc and NYHA Fc. Of 120 patients classified as NYHA Fc I, 43 (35.8%) and 15 (12.5%) were classified as MET Fc II and III, respectively.

### 3.2. HOP Scores in Patients with Different MET Fc

**Figure 1:**
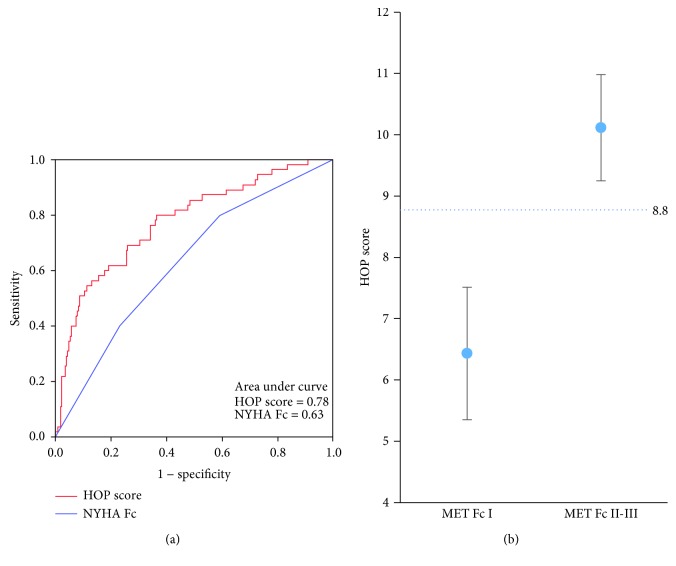
HOP scores in different functional classes as defined by cardiopulmonary exercise test (CPET). (a) The receiver operating characteristic (ROC) curves of the HOP score and the traditional New York Heart Association functional classification (NYHA Fc) in discriminating CPET-defined functional class ≥ II from class I. (b) The HOP score discriminates CPET-defined functional classes II and III from class I with the optimal cutoff value set at 8.8. MET Fc: functional classification defined by metabolic equivalent (MET) as measured by CPET.

HOP scores significantly increased from MET Fc I to III, along with decreased histidine but increased ornithine and phenylalanine (Table 1). ROC curves were conducted to compare the value of HOP scores and NYHA Fc for discriminating patients with MET Fc II and III from those with MET Fc I (Figure 1(a)). The area under the ROC curve of the HOP scores was significantly larger than that of NYHA Fc (0.78 versus 0.63, *p* < 0.001). In addition, based on Youden's index, the optimal cutoff value for HOP scores was set at 8.8 (Figure 1(b)). Of the 58 MET Fc II-III patients diagnosed as NYHA Fc I, 25 (43.1%) had a HOP score of ≥8.8. What a HOP score of ≥8.8 represents was further investigated in patients with HFrEF, HFmrEF, and HFpEF, as follows.

### 3.3. Differences between Patients with HOP Scores ≥ 8.8 and <8.8 in HFrEF, HFmrEF, and HFpEF

Eligibility was assessed in 1041 patients. We excluded 151 patients due to systemic disease (*n* = 16), anticipated survival < 6 months (*n* = 15), being bed ridden (*n* = 33), and creatinine > 3 mg/dl (*n* = 52). The remaining 890 HF outpatients were enrolled and followed up for one year, including 404 HFrEF patients, 168 HFmrEF, and 318 HFpEF. The patients were enrolled in the study a median of 349 days (IQR 88-1128 days) after they were discharged from the hospital. Baseline characteristics of the patients are shown in Table 2. Compared to patients with HFrEF, patients with HFmrEF had higher systolic blood pressure and cholesterol levels and a higher incidence of hypertension, atrial fibrillation, and ischemic etiology but a lower incidence of diuretics use. Patients with HFpEF had higher systolic blood pressure, body mass index, and triglyceride levels, as well as a higher incidence of atrial fibrillation and use of ACEI/ARB but a lower incidence of ischemic etiology.

The prevalence of HOP scores ≥ 8.8 was highest in the HFrEF group (61.4%), followed by the LVmrEF group (42.3%) and the HFpEF group (30.5%). In the HFrEF group, patients with a HOP score ≥ 8.8 were older, more often male, and had higher BNP levels and a wider QRS complex compared to those with a HOP score < 8.8, but they also had lower LVEF and albumin. In the HFmrEF group, those with a HOP score of ≥8.8 were older and had higher BNP levels compared to patients with a HOP score < 8.8, but they also had a lower body mass index, hemoglobin, and incidence of using beta-blockers. In the HFpEF group, those with a HOP score ≥ 8.8, were older, had higher incidence of chronic obstructive pulmonary disease, higher use of diuretics, and higher BNP compared to patients with a HOP score < 8.8 but had lower hemoglobin and incidence of ischemic etiology.

### 3.4. Univariate and Multivariable Analysis

**Figure 2:**
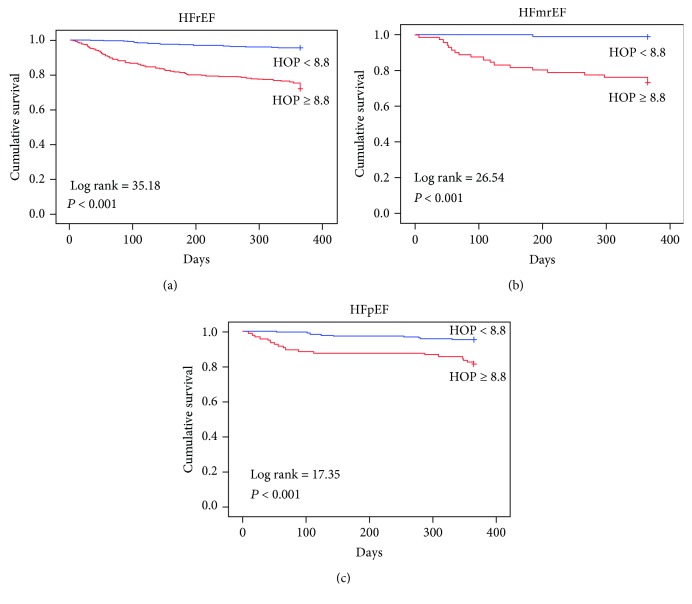
Prognostic value of the HOP score in different populations of heart failure (HF) patients. Kaplan Meier curves for HOP ≥ 8.8 versus <8.8 in HF with reduced ejection fraction (EF) (a), HF with midrange EF (b), and HF with preserved EF (c).

During one-year follow-up, there were 12 (3%) deaths in the HFrEF group, 4 (2.4%) in the HFmrEF group, and 3 (0.9%) in the HFpEF group. The numbers of patients with composite events were 89 (22%) in the HFrEF group, 20 (11.9%) in the HFmrEF group, and 28 (8.8%) in the HFpEF group. The one-year event-free survival for patients with a HOP score of ≥8.8 was 68.1% in the HFrEF group, 73.2% in the HFmrEF group, and 81.4% in the HFpEF group (*p* < 0.001). Univariate analysis shows that the predictors of one-year composite events were HOP score ≥ 8.8, age, albumin, and BNP in the HFrEF group. In the HFmrEF group, predictors were HOP score ≥ 8.8, age, body mass index, and BNP. In the HFmrEF group, predictors of one-year composite events were HOP score ≥ 8.8, age, albumin, and BNP in HFpEF (Table 3). In multivariable analysis, HOP scores ≥ 8.8 remained a one-year composite event predictor independent of other risk factors identified in the univariate analysis. Kaplan-Meier curves show that a HOP score of ≥8.8 significantly identified patients who were at higher risk of composite events in the HFrEF, HFmrEF, and HFpEF groups during the one-year follow-up period (Figure 2).

## 4. Discussion

This study focused on an outpatient population, ordinarily the largest group of patients we face in daily practice and the group that receives the majority of HF care. This population often does not receive optimal care due to inadequate functional assessment and lack of time. Our data show that the traditional NYHA Fc system over- or underestimates functional status. HOP scoring discriminated patients in different MET Fc categories better than NYHA Fc. A HOP score of ≥8.8 was associated with more risk factors for HF events. In addition to functional assessment, a HOP score ≥ 8.8 further identified patients at higher risk of composite events within one year in the HFrEF, HFmrEF, and HFpEF groups, independent of traditional risk factors.

### 4.1. Weakness of Traditional Functional Assessments

Although widely used, the NYHA Fc system has remarkable limitations. Our data showed that it underestimated MET Fc in 34.1% of patients and overestimated it in 44.2% of patients. It is noteworthy that 12.5% of patients classified as NYHA Fc I were actually MET Fc III. Cardiologists' subjective assessment or recognition of patients substantially interferes with NYHA Fc results. Importantly, the criteria committee of the NYHA described the NYHA Fc as “only approximate” and “representative of an expression of the physician's opinion” [7]. A previous interoperator variability study showed only 54% concordance between two cardiologists [4]. Most cardiologists asked patients about their walking ability; however, their self-reported walking status correlated poorly with their actual exercise capacity as measured by CPET. Although CPET is the gold standard, its use is greatly limited by its inconvenience. The reliability of the six min walking test has been shown to be high in patients with stable HF; however, results are affected by a variety of factors unrelated to HF status, including age, sex, height, weight, respiratory disease, peripheral arterial disease, musculoskeletal problems, depression, cognitive impairment, and fear of developing symptoms during the test [24]. By contrast, HOP can be used in patients with limited ability to perform an exercise test and can also avoid most sources of interference that affect NYHA functional classification.

### 4.2. The Value of HOP

Functional capacity actually represents the imbalance between the demand of the body and supply provided by the heart. Previous studies showed that metabolic status, as estimated by metabolomics in plasma, delineated HF metabolic disturbance and provided better prognostic value than BNP and gelactin-3 [8, 9, 16]. After simplifying the complex findings of plasma metabolomics into a HOP score, we noted that HOP retained diagnostic and prognostic value for HF, compensated the weakness of BNP, and was correlated with functional capacity as assessed by six min walk tests [16]. The current study further proves that HOP scores can discriminate functional classifications based on CPET.

The cutoff of 8.8 for HOP scoring not only identified patients with MET Fc ≥ II but also provided value for prognosis. A higher HOP score represented worse prognosis in HFrEF, HFmrEF, and HFpEF. Consistent with our previous studies on HFrEF, HOP scores ≥ 8.8 were related to older patients and higher BNP levels in all three HF populations [16]. In addition, in HFrEF, HOP scores ≥ 8.8 also identified those with a lower LVEF and malnutrition. Compared to HFrEF, in HFmrEF, more patients had atrial fibrillation and ischemic etiology. A HOP score of ≥8.8 in HFmrEF was associated with lower body mass index and hemoglobin, which are well known as factors in poor prognosis. Similar to HFmrEF, HFpEF also entailed a higher incidence of atrial fibrillation than HFrEF. Furthermore, HOP scores of ≥8.8 were associated with a higher incidence of lung disease and using diuretics but with lower hemoglobin, suggesting more comorbidities and more severe symptoms.

It is also interesting to consider the mechanisms that account for decreased histidine but increased phenylalanine and ornithine, which were correlated with functional classification. Previous studies have shown that increased phenylalanine levels are associated with increased muscular protein breakdown, nitric oxide synthesis dysregulation, and tetrahydrobiopterin depletion [16, 25–27]. To produce energy for cardiac tissues, histidine is converted to glutamate and enters the glutamate-ornithine-proline pathway or the Krebs cycle [8, 16]. Lower histidine levels can represent substantial histidine deficiency, since a large amount of histidine pools in hemoglobin and carnosine in the muscle [28]. Increased ornithine, an important component of the urea cycle, also indicates overload handling of the waste from using amino acids from muscular protein as an energy source. These mechanisms explain the tight link between HOP score and functional status.

### 4.3. Study Limitations

A few limitations are to be noted in this study. First, our data showed a significant trend revealing that a higher proportion of patients classed as MET Fc III was female. This could be caused by sex-related performance on CPET. However, metabolism-based assessment may be a substitute. Future studies may investigate how to apply HOP scores to different sexes. Second, our analyses are based on data from patients who are able to perform CPET. Whether the findings noted in this study can be applied to those unable to perform CPET requires further study. Finally, the correlation between the HOP score and METs was established based on a cohort of chronic HF patients. The value of HOP scores in patients with acute HF remains to be explored.

## 5. Conclusions

The traditional NYHA Fc assessment system under- and overestimates the MET Fc of a substantial number of HF outpatients. An amino acid-based metabolic profile (HOP score) provides a measure of the metabolic status that correlates with the functional status as estimated by CPET better than the NYHA Fc system. A HOP score of ≥8.8 identifies HF patients in the functional class ≥ II and at risk of HF-related events. Our data also proves that the HOP score ≥ 8.8 has prognostic value not only in HFrEF but also for patients with HFmrEF and HFpEF, providing evidence that patients with the functional class ≥ II warrant aggressive treatment. The value of utilizing the HOP score as a follow-up assessment tool merits further study.

## Figures and Tables

**Table 1:** Demographic and laboratory data in heart failure patients in different functional classes (Fc) as defined by cardiopulmonary exercise testing.

	MET Fc I	MET Fc II	MET Fc III	
	*n* = 135	*n* = 148	*n* = 104	*p* for trend∗
Age (years)	52.0 ± 10.5	60.0 ± 13.3	65.1 ± 10.3	<0.001
Male (%)	126 (93.3)	105 (70.9)	55 (52.9)	<0.001
LVEF (%)	42.6 ± 17.9	42.7 ± 16.8	35.8 ± 12.9	0.002
NYHA functional classification				<0.001
I (%)	62 (45.9)	43 (29.1)	15 (14.4)	
II (%)	46 (34.1)	60 (40.5)	29 (27.9)	
III (%)	27 (20)	45 (30.4)	60 (57.7)	
Blood pressure (mmHg)				
Systolic	121 ± 17.6	122 ± 20.7	121 ± 21.4	0.888
Diastolic	73.5 ± 11.4	72.3 ± 12.5	70.6 ± 12.5	0.073
Heart rate (beats/min)	71.7 ± 16.2	81.5 ± 18.2	81.1 ± 16.8	<0.001
Comorbidity				
Diabetes mellitus (%)	41 (30.4)	43 (29.1)	61 (58.7)	<0.001
Hypertension (%)	77 (57)	95 (64.2)	61 (58.7)	0.724
Atrial fibrillation (%)	24 (17.8)	48 (32.4)	17 (16.3)	0.984
COPD (%)	10 (7.4)	16 (10.8)	10 (9.6)	0.519
Ischemia (%)	69 (51.1)	82 (55.4)	79 (76)	<0.001
Body mass index (kg/m^2^)	25.8 ± 3.81	25.7 ± 4.54	24.0 ± 4.99	0.003
Medication				
ACEI or ARB (%)	135 (100)	146 (98.6)	103 (99)	0.358
*β*-Blocker (%)	130 (96.3)	144 (97.3)	98 (94.2)	0.459
Diuretic (%)	56 (41.5)	81 (54.7)	62 (59.6)	0.004
Laboratory data				
BNP (log)	1.98 ± 0.78	2.44 ± 0.62	2.64 ± 0.58	<0.001
Cholesterol (mg/dl)	192 ± 41.6	180 ± 41.5	182 ± 37.8	0.056
Triglyceride (mg/dl)	143 ± 87.3	152 ± 107.6	146 ± 94.0	0.845
Serum sodium (mEq/l)	140 ± 2.36	139 ± 10.7	140 ± 3.99	0.942
Hemoglobin (g/dl)	14.4 ± 1.31	13.4 ± 2.05	12.9 ± 2.27	<0.001
Albumin (g/dl)	4.3 ± 0.46	4.1 ± 0.44	4.0 ± 0.47	<0.001
eGFR (ml/min/1.73 m^2^)	81.6 ± 19.2	73.1 ± 25.7	56.1 ± 27.1	<0.001
QRS complex (msec)	100 ± 16.6	102 ± 21.4	118 ± 32.9	<0.001
Metabolic equivalent (MET)	6.67 ± 0.86	4.71 ± 0.49	3.36 ± 0.51	<0.001
HOP score	6.43 ± 6.36	9.06 ± 6.56	11.6 ± 7.32	<0.001
Histidine (*μ*M)	81.2 ± 13.9	75.8 ± 13.8	72.6 ± 12.8	<0.001
Ornithine (*μ*M)	89.5 ± 26.6	92.8 ± 38.5	106 ± 36.7	<0.001
Phenylalanine (*μ*M)	63.3 ± 11.0	63.6 ± 11.6	70.6 ± 17.8	<0.001

ACEI: angiotensin-converting enzyme inhibitor; ARB: angiotensin receptor blocker; COPD: chronic obstructive pulmonary disease; eGFR: estimated glomerular filtration rate; HF: heart failure; HOP: histidine, ornithine, and phenylalanine; LVEF: left ventricular ejection fraction; MET Fc: functional classification defined by metabolic equivalent (MET) measured by cardiopulmonary exercise testing; NYHA: New York Heart Association. ∗Comparison of patients from Fc I to Fc III.

**Table 2:** Differences between patients with HOP scores ≥ 8.8 and HOP scores < 8.8 in HFrEF, HFmrEF, and HFpEF patients.

	HFrEF			HFmrEF			HFpEF			
	All	HOP < 8.8	HOP ≥ 8.8	All	HOP < 8.8	HOP ≥ 8.8	All	HOP < 8.8	HOP ≥ 8.8	*p* value^a^
	*n* = 404	*n* = 156	*n* = 248	*n* = 168	*n* = 97	*n* = 71	*n* = 318	*n* = 221	*n* = 97	
Age (years)	61.4 ± 13.1	58.9 ± 12.4	63.0 ± 13.3∗	63.9 ± 11.5	60.4 ± 10.3	68.7 ± 11.4^#^	63.0 ± 14.4	60.6 ± 14.3	68.6 ± 13.2^#^	0.078
Male (%)	303 (75)	101 (64.7)	202 (81.5)^#^	120 (71.4)	66 (68)	54 (76.1)	223 (70.1)	149 (67.4)	74 (76.3)	0.322
LVEF (%)	27.6 ± 8.47	29.1 ± 8.19	26.7 ± 8.52^#^	44.2 ± 2.98	44.5 ± 3.12	43.9 ± 2.77	61.31 ± 7.34	60.9 ± 7.28	62.4 ± 7.40	<0.001
Blood pressure (mmHg)										
Systolic	117 ± 16.9	118 ± 17.1	116 ± 16.8	121 ± 18.2^†^	122 ± 19.1	120 ± 17.0	124 ± 15.8^‡^	124 ± 14.8	123 ± 18.0	<0.001
Diastolic	73.7 ± 11.3	74.7 ± 11.3	73.1 ± 11.3	74.5 ± 11.7	75.5 ± 12.2	73.1 ± 10.7	75.8 ± 12.2	76.5 ± 12.1	74.2 ± 12.3	0.060
Heart rate (beats/min)	76.6 ± 33.9	76.8 ± 35.6	76.5 ± 32.9	73.9 ± 33.3	74.5 ± 32.9	73.2 ± 34.1	74.4 ± 37.6	76.7 ± 36.2	69.3 ± 40.5	0.602
Comorbidities (%)										
Diabetes mellitus	177 (43.8)	61 (39.1)	116 (46.8)	84 (50)	46 (47.4)	38 (53.5)	133 (41.8)	89 (40.3)	44 (45.4)	0.219
Hypertension	250 (61.9)	98 (62.8)	152 (61.3)	122 (72.6)^†^	69 (71.1)	53 (74.6)	212 (66.7)	148 (67)	64 (66)	0.043
Atrial fibrillation	68 (16.8)	22 (14.1)	46 (18.5)	45 (26.8)^‡^	31 (32)	14 (19.7)	98 (30.8)^‡^	61 (27.6)	37 (38.1)	<0.001
COPD	30 (7.4)	13 (8.3)	17 (6.9)	16 (9.5)	6 (6.2)	10 (14.1)	24 (7.5)	9 (4.1)	15 (15.5)^#^	0.674
Ischemia	228 (56.4)	87 (55.8)	141 (56.9)	118 (70.2)^‡^	64 (66)	54 (76.1)	135 (42.5)^‡^	108 (48.9)	27 (27.8)^#^	<0.001
Body mass index (kg/m^2^)	24.9 ± 4.26	25.4 ± 3.84	24.6 ± 4.48	25.5 ± 4.74	26.6 ± 4.87	24.0 ± 4.12^#^	25.8 ± 4.25^†^	25.5 ± 4.10	25.9 ± 5.58	0.023
Medication										
ACEI or ARB (%)	367 (90.8)	140 (89.7)	227 (91.5)	149 (88.7)	89 (91.8)	60 (84.5)	303 (95.3)^†^	214 (96.8)	89 (91.8)	0.019
*β*-Blocker (%)	350 (86.6)	134 (85.9)	216 (87.1)	152 (90.5)	92 (94.8)	60 (84.5)∗	281 (88.4)	198 (89.6)	83 (85.6)	0.422
Diuretic (%)	250 (61.9)	92 (59)	158 (63.7)	80 (47.6)^‡^	49 (50.5)	31 (43.7)	175 (55)	107 (48.4)	68 (70.1)^#^	0.005
Laboratory data										
BNP (log)	2.7 ± 0.57	2.4 ± 0.58	2.9 ± 0.48^#^	2.3 ± 0.66^‡^	2.1 ± 0.59	2.6 ± 0.67^#^	1.9 ± 0.70^‡^	1.7 ± 0.61	2.4 ± 0.69^#^	<0.001
Cholesterol (mg/dl)	179 ± 45.6	182 ± 40.6	176 ± 48.5	192 ± 66.9^†^	195 ± 69.2	188 ± 63.9	189 ± 47.3^†^	193 ± 46.1	180 ± 48.8	0.003
Triglyceride (mg/dl)	118 ± 63.1	123 ± 66.2	116 ± 61.0	131 ± 95.2	137 ± 106.2	124 ± 77.9	140 ± 95.3^‡^	142 ± 92.7	135 ± 101.3	0.002
Serum sodium	139 ± 2.9	139 ± 2.9	139 ± 2.9	139 ± 3.3	138 ± 3.6	139 ± 2.7	139 ± 3.1	139 ± 3.1	139 ± 3.3	0.102
Hemoglobin (g/dl)	13.3 ± 1.9	13.0 ± 2.0	13.0 ± 1.8	13.4 ± 2.2	13.8 ± 2.2	12.9 ± 2.04^#^	13.7 ± 2.1	13.9 ± 2.12	13.2 ± 1.9^#^	0.071
Albumin (g/dl)	3.8 ± 0.53	3.9 ± 0.44	3.7 ± 0.58^#^	3.9 ± 0.52	3.9 ± 0.42	3.8 ± 0.63	3.8 ± 0.43	3.8 ± 0.45	3.8 ± 0.40	0.093
eGFR (ml/min/1.73 m^2^)	71 ± 26.3	73 ± 25.4	69 ± 26.8	73 ± 33.6	76 ± 24.0	69 ± 43.2	73 ± 24.4	73.6 ± 24.1	71.9 ± 25.2	0.416
QRS complex (msec)	114 ± 28.7	110 ± 28.3	117 ± 28.7∗	106 ± 22.1^‡^	105 ± 20.6	108 ± 24.1	99 ± 24.2^‡^	98.6 ± 22.1	100.7 ± 28.4	<0.001
HOP score	10.5 ± 7.42	3.6 ± 4.37	14.8 ± 5.41^#^	8.1 ± 6.29^‡^	3.9 ± 3.50	13.9 ± 4.25^#^	6.5 ± 6.19^‡^	3.4 ± 4.10	13.6 ± 3.86^#^	<0.001
Histidine (*μ*M)	74.2 ± 14.1	80.1 ± 12.3	70.5 ± 14.0^#^	75.0 ± 14.1	81.9 ± 10.6	67.9 ± 14.3^#^	79.9 ± 14.2^‡^	83.6 ± 12.6	71.5 ± 14.1^#^	<0.001
Ornithine (*μ*M)	101 ± 40.9	85.8 ± 26.5	111 ± 45.24^#^	97.4 ± 30.8	87.6 ± 20.1	111 ± 37.4^#^	91.6 ± 30.9^‡^	83.8 ± 24.7	109 ± 36.0^#^	0.002
Phenylalanine (*μ*M)	67.2 ± 15.9	59.4 ± 10.3	72.1 ± 16.8^#^	65.2 ± 13.6	61.7 ± 9.8	69.9 ± 16.5^#^	64.5 ± 12.5^†^	62.3 ± 9.94	69.6 ± 15.8^#^	0.034

ACEI: angiotensin-converting enzyme inhibitor; ARB: angiotensin receptor blocker; COPD: chronic obstructive pulmonary disease; HF: heart failure; HOP: histidine, ornithine, and phenylalanine; LVEF: left ventricular ejection fraction; LVrEF: LVEF < 40%, LVmrEF: LVEF = 40%−49%, LVpEF: LVEF ≥ 50%.∗*p* < 0.05 and ^#^*p* < 0.01, compared to patients with HOP score < 8.8. ^†^*p* < 0.05 and ^‡^*p* < 0.01, compared to patients with LVrEF. ^a^ANOVA *p* value to compare LVrEF, LVmrEF, and LVpEF.

**Table 3:** COX univariate and multivariable analysis in HFrEF, HFmrEF, and HFpEF patients.

	HFrEF		HFmrEF		HFpEF	
	Univariate HR (95% CI)	Multivariable HR (95% CI)	Univariate HR (95% CI)	Multivariable HR (95% CI)	Univariate HR (95% CI)	Multivariable HR (95% CI)
HOP score ≥ 8.8	5.78 (2.99-11.16)^†^	3.80 (1.56-9.25)^†^	29.95 (4.01-103)^†^	9.60 (1.12-62.3)^∗^	4.48 (2.07-9.70)^†^	3.05 (1.09-8.57)^∗^
Age (years)	1.03 (1.01-1.45)^†^	1.02 (1.01-1.04)^∗^	1.05 (1.01-1.09)^∗^	0.97 (0.92-1.02)	1.04 (1.01-1.07)^∗^	1.01 (0.97-1.05)
Sex	1.46 (0.86-2.48)		1.18 (0.43-3.25)		1.59 (0.65-3.93)	
LVEF (%)	0.99 (0.96-1.01)		0.85 (0.72-1.01)		0.99 (0.94-1.04)	
Hypertension	1.38 (0.88-2.15)		3.62 (0.84-15.58)		39.9 (1.56-102)	
Diabetes mellitus	1.02 (0.67-1.55)		2.40 (0.92-6.25)		1.90 (0.90-4.03)	
COPD	0.70 (0.28-1.73)		0.49 (0.067-3.67)		2.17 (0.75-6.26)	
Ischemic	1.35 (0.88-2.07)		0.76 (0.30-1.91)		0.64 (0.29-1.41)	
BMI (kg/m^2^)	0.97 (0.92-1.02)		0.84 (0.75-0.93)^†^	0.93 (0.82-1.05)	0.98 (0.90-1.07)	
Cholesterol (mg/dl)	1.00 (0.99-1.01)		1.00 (0.99-1.01)		1.00 (0.99-1.01)	
Triglyceride (mg/dl)	1.00 (0.99-1.01)		1.00 (0.99-1.01)		1.00 (0.99-1.01)	
Serum sodium (mEq/l)	0.99 (0.99-1.00)		0.99 (0.99-1.00)		1.01 (0.99-1.03)	
Albumin (g/dl)	0.36 (0.25-0.52)^†^	0.41 (0.25-0.65)^†^	0.46 (0.20-1.06)		0.37 (0.16-0.83)^∗^	0.40 (0.17-0.95)^∗^
QRS (msec)	1.00 (0.99-1.01)		0.99 (0.98-1.01)		0.99 (0.98-1.00)	
BNP (log)	3.84 (2.26-6.53)^†^	2.02 (1.16-3.50)^∗^	8.27 (2.30-29.66)^†^	3.36 (1.02-11.0)^∗^	2.15 (1.04-4.46)^∗^	1.47 (0.63-3.43)

^∗^ *p* < 0.05 and ^†^*p* < 0.01 in the COX univariate and multivariable analysis. CI: confidence interval; COPD: chronic obstructive pulmonary disease; HF: heart failure; HOP: histidine, ornithine, and phenylalanine; HR: hazard ratio; LVEF: left ventricular ejection fraction; LVrEF: LVEF < 40%; LVmrEF: LVEF = 40%−49%; LVpEF: LVEF ≥ 50%.

## Data Availability

The data that support the findings of this study are available from the corresponding author on reasonable request.

## References

[1] McMurray J. J. V., Packer M., Desai A. S. (2014). Angiotensin–neprilysin inhibition versus enalapril in heart failure. *New England Journal of Medicine*.

[2] Zannad F., McMurray J., Krum H. (2011). Eplerenone in patients with systolic heart failure and mild symptoms. *New England Journal of Medicine*.

[3] Lucas C., Stevenson L. W., Johnson W. (1999). The 6-min walk and peak oxygen consumption in advanced heart failure: aerobic capacity and survival. *American Heart Journal*.

[4] Raphael C., Briscoe C., Davies J. (2007). Limitations of the New York Heart Association functional classification system and self-reported walking distances in chronic heart failure. *Heart*.

[5] Dunagan J., Adams J., Cheng D. (2013). Development and evaluation of a treadmill-based exercise tolerance test in cardiac rehabilitation. *Baylor University Medical Center Proceedings*.

[6] Jetté M., Sidney K., Blümchen G. (1990). Metabolic equivalents (METS) in exercise testing, exercise prescription, and evaluation of functional capacity. *Clinical Cardiology*.

[7] Criteria Committee (1964). *Diseases of the Heart and Blood Vessels. Nomenclature and Criteria for Diagnosis*.

[8] Cheng M. L., Wang C. H., Shiao M. S. (2015). Metabolic disturbances identified in plasma are associated with outcomes in patients with heart failure: diagnostic and prognostic value of metabolomics. *Journal of the American College of Cardiology*.

[9] Wang C. H., Cheng M. L., Liu M. H., Kuo L. T., Shiao M. S. (2017). Metabolic profile provides prognostic value better than galectin-3 in patients with heart failure. *Journal of Cardiology*.

[10] Tang H. Y., Wang C. H., Ho H. Y. (2018). Lipidomics reveals accumulation of the oxidized cholesterol in erythrocytes of heart failure patients. *Redox Biology*.

[11] Hunter W. G., Kelly J. P., McGarrah R. W., Kraus W. E., Shah S. H. (2016). Metabolic dysfunction in heart failure: diagnostic, prognostic, and pathophysiologic insights from metabolomic profiling. *Current Heart Failure Reports*.

[12] Alexander D., Lombardi R., Rodriguez G., Mitchell M. M., Marian A. J. (2011). Metabolomic distinction and insights into the pathogenesis of human primary dilated cardiomyopathy. *European Journal of Clinical Investigation*.

[13] Tenori L., Hu X., Pantaleo P. (2013). Metabolomic fingerprint of heart failure in humans: a nuclear magnetic resonance spectroscopy analysis. *International Journal of Cardiology*.

[14] Wang C. H., Cheng M. L., Liu M. H. (2016). Increased p-cresyl sulfate level is independently associated with poor outcomes in patients with heart failure. *Heart Vessels*.

[15] Würtz P., Havulinna A. S., Soininen P. (2015). Metabolite profiling and cardiovascular event risk: a prospective study of 3 population-based cohorts. *Circulation*.

[16] Wang C. H., Cheng M. L., Liu M. H. (2018). Amino acid-based metabolic panel provides robust prognostic value additive to B-natriuretic peptide and traditional risk factors in heart failure. *Disease Markers*.

[17] Wang C. H., Cheng M. L., Liu M. H. (2018). Simplified plasma essential amino acid-based profiling provides metabolic information and prognostic value additive to traditional risk factors in heart failure. *Amino Acids*.

[18] Ponikowski P., Voors A. A., Anker S. D. (2016). 2016 ESC guidelines for the diagnosis and treatment of acute and chronic heart failure: the task force for the diagnosis and treatment of acute and chronic heart failure of the European Society of Cardiology (ESC)developed with the special contribution of the Heart Failure Association (HFA) of the ESC. *European Journal of Heart Failure*.

[19] Maisel A. S., Clopton P., Krishnaswamy P. (2004). Impact of age, race, and sex on the ability of B-type natriuretic peptide to aid in the emergency diagnosis of heart failure: results from the Breathing Not Properly (BNP) multinational study. *American Heart Journal*.

[20] Pappa-Louisi A., Nikitas P., Agrafiotou P., Papageorgiou A. (2007). Optimization of separation and detection of 6-aminoquinolyl derivatives of amino acids by using reversed-phase liquid chromatography with on line UV, fluorescence and electrochemical detection. *Analytica Chimica Acta*.

[21] Frank M. P., Powers R. W. (2007). Simple and rapid quantitative high-performance liquid chromatographic analysis of plasma amino acids. *Journal of Chromatography B*.

[22] Miyamara M., Honda Y. (1972). Oxygen intake and cardiac output during maximal treadmill and bicycle exercise. *Journal of Applied Physiology*.

[23] Williford H. N., Sport K., Wang N., Olson M. S., Blessing D. (1994). The prediction of fitness levels of United States Air Force officers: validation of cycle ergometry. *Military Medicine*.

[24] Demers C., McKelvie R., Negassa A., Yusuf S., RESOLVD Pilot Study Investigators (2001). Reliability, validity, and responsiveness of the six-minute walk test in patients with heart failure. *American Heart Journal*.

[25] Nishijima Y., Sridhar A., Bonilla I. (2011). Tetrahydrobiopterin depletion and NOS2 uncoupling contribute to heart failure-induced alterations in atrial electrophysiology. *Cardiovascular Research*.

[26] Ziolo M. T., Maier L. S., Piacentino V., Bossuyt J., Houser S. R., Bers D. M. (2004). Myocyte nitric oxide synthase 2 contributes to blunted *β*-adrenergic response in failing human hearts by decreasing Ca^2+^ transients. *Circulation*.

[27] Aquilani R., la Rovere M. T., Febo O. (2012). Preserved muscle protein metabolism in obese patients with chronic heart failure. *International Journal of Cardiology*.

[28] Kriengsinyos W., Rafii M., Wykes L. J., Ball R. O., Pencharz P. B. (2002). Long-term effects of histidine depletion on whole-body protein metabolism in healthy adults. *The Journal of Nutrition*.

